# Are Migraine With and Without Aura Really Different Entities?

**DOI:** 10.3389/fneur.2019.00982

**Published:** 2019-10-02

**Authors:** Zsigmond Tamás Kincses, Dániel Veréb, Péter Faragó, Eszter Tóth, Krisztián Kocsis, Bálint Kincses, András Király, Bence Bozsik, Árpád Párdutz, Délia Szok, János Tajti, László Vécsei, Bernadett Tuka, Nikoletta Szabó

**Affiliations:** ^1^Department of Neurology, Faculty of Medicine, Interdisciplinary Excellent Centre, University of Szeged, Szeged, Hungary; ^2^Department of Radiology, University of Szeged, Szeged, Hungary; ^3^Brain and Mind Research, Central European Institute of Technology, Brno, Czechia; ^4^MTA-SZTE, Neuroscience Research Group, Szeged, Hungary

**Keywords:** DTI, functional MRI, microstructure, migraine with and without aura, pathomechanism

## Abstract

**Background:** Migraine research is booming with the rapidly developing neuroimaging tools. Structural and functional alterations of the migrainous brain were detected with MRI. The outcome of a research study largely depends on the working hypothesis, on the chosen measurement approach and also on the subject selection. Against all evidence from the literature that migraine subtypes are different, most of the studies handle migraine with and without aura as one disease.

**Methods:** Publications from PubMed database were searched for terms of “migraine with aura,” “migraine without aura,” “interictal,” “MRI,” “diffusion weighted MRI,” “functional MRI,” “compared to,” “atrophy” alone and in combination.

**Conclusion:** Only a few imaging studies compared the two subforms of the disease, migraine with aura, and without aura, directly. Functional imaging investigations largely agree that there is an increased activity/activation of the brain in migraine with aura as compared to migraine without aura. We propose that this might be the signature of cortical hyperexcitability. However, structural investigations are not equivocal. We propose that variable contribution of parallel, competing mechanisms of maladaptive plasticity and neurodegeneration might be the reason behind the variable results.

## Introduction

Migraine is a heterogeneous disease affecting cca 10–20% of the population worldwide. It is associated with significant disability, reduced quality of life, and consequently poses an enormous financial burden to society (1). Since migraine ranks among the top disorders causing disability (2). It is in the focus of neuroimaging, molecular, and pharmaceutical research. In 20% of the cases, migraine headache is preceded or accompanied by reversible focal neurological symptoms, such as visual, motor, sensory, or speech disturbances (3). The ICHD-3 classification (4) bases the diagnosis of migraine on the patient's medical history and physical examination. Accordingly, migraine can be categorized into migraine with aura (MWA) and without aura (MWoA) as subtypes of the disease (among other categories). Besides the similarities in the epidemiology, clinical presentation, and the genetic evidence that MWA and MWoA largely overlap (5), the question has been raised a few years ago: are MWA and MWoA separate entities (6–8) or rather the two ends of a spectrum? Nevertheless, studying mixed groups of migraine patients should be avoided in further investigations if critical differences exist. Nevertheless, studying mixed groups of migraine patients should be avoided in further investigations if critical differences exist.

Since magnetic resonance imaging (MRI) makes it possible to investigate the structure and function of the brain *in vivo*, hundreds of papers were published in the last 20 years that describe the migrainous brain using neuroimaging methodology. The majority of these studies examined mixed patient groups or compared only one subtype to healthy individuals.

This review article summarizes the most significant neuroimaging results from studies comparing MWA patients to MWoA. To identify relevant articles, we searched the PubMed database for terms of “migraine with aura,” “migraine without aura,” “interictal,” “MRI,” “diffusion weighted MRI” (DWI), “functional MRI” (fMRI), “compared to,” “atrophy” alone and in combination up to June 2019.

## Results from Functional Neuroimaging

Brain activity during rest and task performance can be described non-invasively by measuring the blood oxygen level dependent (BOLD) signal with functional MRI. Traditional fMRI studies compare signal differences in various phases of a task, but recently, there has also been a growing interest in studying brain activity patterns during rest. Interestingly, remote areas show synchronous activity, which renders resting state activity into functional networks (9, 10). Although fMRI parameters remain basically the same, we are witnessing a rapid development in the statistical analysis of fMRI scans.

Two publications confirmed brain activation differences between MWA and MWoA in the interictal phase (11, 12). Datta et al. (11) described higher BOLD response in migraine in response to visual stimuli in a BOLD fMRI study. This higher BOLD response was more robust in MWA than in MWoA patients. Interestingly the resting perfusion parameters of the two groups was not different, hence the authors discussed their finding in the light of the existing evidences that it relates to hyperresponsiveness of the visual cortex in MWA. On the contrary, resting brain perfusion did not differ between patients and controls or between MWA and MWoA patients (11). In a considerably larger cohort Cucchiara et al. found similarly greater BOLD amplitude in the visual cortex in MWA that positively correlated with visual discomfort score. No such correlation was found in MWoA (12).

Limited data are available on resting brain activity. Increased expression of the visual resting functional network was found in MWA compared to MWoA and controls (13). In our earlier investigation we found higher amplitude of resting state activity fluctuation in all identified resting state networks in the 0.08–0.04 Hz frequency range in MWA as compared to MWoA (14). On the contrary, lower amplitudes were found in the default mode network in MWoA compared to controls.

Reduced connectivity between the occipital lobe and anterior insula was found in MWA but not in MWoA, and the connectivity strength correlated with migraine severity in MWA (15). Increased connectivity was found in the default mode network in the pre-central gyrus, post-central gyrus, insular cortex, angular gyrus, supramarginal gyrus in MWA compared to MWoA (16).

## Results from Structural Neuroimaging

### Cortical Thickness

Several investigations have shown that there are gray matter alterations in migraine: the gray matter density of several pain related cortical regions is reduced compared to healthy individuals (17). It should be noted that similar brain structural alterations were found in other chronic pain conditions. Importantly, only a few investigations concentrated on comparing the two subgroups of migraine.

Granziera et al. found increased cortical thickness and altered microstructure in migraineurs in the white matter beneath motion processing areas, namely motion processing visual areas and V3A area, but there were no differences between MWA and MWoA patients (18). In a similar cohort, voxel-based morphometry (VBM) did not detect any differences between the two patient groups (13). In a multicentre study involving a considerably larger migraine population, MWoA patients exhibited thinner cortex in the left central sulcus, in the left occipito-temporal gyrus, in the right cuneus and the superior parietal gyrus bilaterally. In some of these regions, the cortical thickness correlated with the frequency of migraine attacks and disease duration (19). Interestingly, a few of these regions were not only thinner in MWoA as compared to controls, but also when compared to MWA.

### Diffusion Tensor Imaging

Among the structural abnormalities, white matter microstructure changes, as described by DWI, are receiving more and more attention. DWI is sensitive to the diffusion of water molecules, which in the brain is largely restricted by the membranes of cellular and sub-cellular elements. By fitting a diffusion tensor model it is possible to estimate diffusion parameters that reflect the microscopic organization of the measured volume (20).

White matter microstructural changes in MWA were reported, but studies are not congruent in calculated diffusion parameters and results. DaSilva et al. presented lower fractional anisotropy (FA) in the ventral trigemino-thalamic pathway in MWA and lower FA was detected in the ventrolateral periaqueductal gray matter (PAG) in MWoA (21). No correlation was found with clinical parameters. While migraineurs showed reduced FA subjacent to visual motion processing areas, no differences in diffusion parameters were found between MWA and MWoA (18). Similarly, tract-based-spatial statistics (TBSS) and a pre-defined region-of-interest analysis from fMRI results did not reveal microstructural white matter alterations between the two subtypes (13, 22). On the other hand, we found extensive white matter regions showing higher FA in MWA in a whole brain TBSS analysis (8). Also, we found that clinical parameters, such as disease duration and estimated lifetime attack number were associated with lower axial diffusivity (AD) in the left superior longitudinal fascicle, the left corticospinal tract and with the right superior longitudinal fascicle of MWA patients (8).

## Discussion

There are only few neurological disorders that were investigated so extensively and the hypotheses for its pathophysiology went through such evolution like migraine. In spite of this enormous body of research, the “migraine puzzle” is still incomplete. While migraine was thought to be a functional disease of the brain, recent studies have shown that brain structure and microstructure also exhibit profound alterations. Independent MRI studies observed functional and structural differences between MWA and MWoA in the interictal period. In summary, it can be pointed out that studies concur in finding higher brain activity/activation in MWA, but structural differences between the two subtypes of the disease are not so well-established, and results are ambiguous in the literature (Table 1).

**Table 1:** Structural and functional MRI studies comparing migraine without to migraine with aura.

**Method**	**Subjects**	**Main findings**
BOLD fMRI; resting ASL Datta et al. (11)	25 MWA, 25 MwoA, 25 controls	Robust visual pathway activation was in MWA. ASL showed no difference.
BOLD fMRI Cucchiara et al. (12)	51 MWA, 45 MwoA, 45 controls	Greater visual cortex activation and correlation with light sensitivity in MWA.
RSN fMRI; DTI Tedeschi et al. (13)	20 MWA, 20 MwoA, 20 controls	Increased component activity was in lingual gyrus from visual network in MWA. Structural analysis showed no differences.
RSN fMRI; T1 Niddam et al. (15)	26 MWA, 26 MwoA, 26 controls	Reduced connectivity was between visual cortex and insula in MWA. The right parahippocampal region was decreased in MWA.
RSN fMRI Lo Buono (16)	14 MWA, 14 MwoA, 14 controls	Increased functional connectivity was in angular gyrus, supramarginal gyrus, pre-central gyrus, post-central gyrus, insular cortex in MWA.
RSN fMRI Faragó et al. (14)	18 MWA, 33 MwoA, 32 controls	Amplitude of RSN fluctuation is higher in MWA: cingulate cortex, superior parietal lobule, cerebellum and bilateral frontal regions.
DTI Tessitore et al. (22)	20 MWA, 20 MwoA, 20 controls	TBSS and VBM analyses detected no differences.
DTI, T1 DaSilva et al. (21)	12 MWA, 12 MwoA, 12 controls	Trigeminothalamic tract and periaqueductal gray area showed difference in FA.
DTI Granziera et al. (18)	12 MWA, 12 MwoA, 15 controls	White matter analysis and cortical thickening showed no differences.
T1 Magon et al. (19)	38 MWA, 93 MwoA, 115 controls	MWoA showed thinner cortex: left central sulcus, left occipito-temporal gyrus, right cuneus, bilateral superior parietal gyrus; MWoA showed thicker cortex: inferior temporal gyrus.
DTI Szabó et al. (8)	18 MWA, 25 MwoA, 28 controls	FA was higher in left parieto-occipital white matter in MWA. Clinical parameters correlated with white matter integrity in MWA.

In MWA, during the transient visual, sensory or language symptoms a slow depolarization wave called cortical spreading depression (CSD) spreads through the cortex (23). During visual aura, BOLD signal changes develop in the occipital cortex and progress slowly, reflecting underlying depolarization waves (24). Apart from being the putative cause of the aura symptoms, CSD has been associated with neuroinflammation, possibly contributing further to the headache by activating the meningeal nociceptors and the neurons in the spinal trigeminal nucleus and trigeminal nucleus caudalis (25, 26). Neurophysiological investigations showed that the two subtypes of the disease differ considerably. The amplitudes of visual evoked potentials (VEP) were higher in migraineurs (27–30). Recent reports showed that hyperexcitability, as measured via VEP is predominantly true for MWA (31, 32). The threshold of transcranial magnetic stimulation (TMS) evoked phosphenes is also lower in migraineurs and the prevalence of phosphenes is higher (33). Interestingly, a recent metaanalysis pointed out that, similarly to the VEP results, this kind of TMS measured hyperexcitability is only true for patients experiencing aura (34). Moreover, the perception of cross-modal interaction that depends highly on cortical hyperexcitability differs between healthy volunteers and migraineurs (35). The effect is more pronounced in MWA. A possible background mechanism behind this hyperexcitability might be the altered neurochemical milieu, the imbalance of the excitatory and inhibitory neurotransmitter levels in migraineurs as detected by MR spectroscopy or other neurochemical approaches [e.g., glutamate and GABA, see (36, 37) for a review]. However, no study investigated the differences between MWA and MWoA. The above-mentioned functional imaging studies also demonstrated higher activation/activity in MWA. Considering that hyperexcitability comes along with increased firing frequency (38) that has a higher energy demand, it easily follows that BOLD fMRI studies find increased amplitude of response or resting activity fluctuation. In consequence to this increased activity, especially if it is regionally specific, interregional connections might strengthen, that could be measured as increased functional connectivity.

Nevertheless, one should not forget that fMRI is measuring the indirect vascular response to neuronal activity/activation. Since migraine is a neurovascular disease the identified differences in any fMRI study might be due to the filtering effect of the altered hemodynamic response function. In fact, altered vasomotor reactivity was identified in MWA (39).

The results of structural investigations are far less equivocal about the differences between the two subtypes of the disease.

A prominent reason behind the differences in the outcome of the structural studies might be the pathomechanism itself. Structural alterations could either be a (1) consequence or the (2) cause of the disease. In case of the former, one might consider two alternatives:

- The recurring painful attacks and hyperexcitability could lead to maladaptive plasticity. Use-dependent plasticity induced morphological changes are well-known in the gray and white matter (40–42). Repeated pain stimuli can also increase gray matter density in pain processing regions including the cingulate and the contralateral somatosensory cortex (43). One might hypothesize that the increased firing frequency due to hyperexcitability may induce similar use-dependent plastic changes. And finally, plastic changes were reported in animals after induction of CSD (44, 45). These processes presumably appear in the form of increased FA and thickened cortex (40–42).
- The underlying pathology might also cause degenerative processes in migraine. CSD might well-contribute to the noxious process (46), as it induces neuroinflammation and cellular damage (47–49). The recurring painful attacks and the cortical hyperexcitability might lead to excessive glutamate release (50), which is also known to induce excitotoxicity and cell death (51). CSD causes upregulation of matrix-metalloproteases (MMP) (52) and increased MMP activity was described in human migraineurs (53). This can lead to the leakage of the blood-brain barrier and inflammatory response and neuronal damage (54). In line with these hypotheses, increased ictal levels of S100B (a marker of glial damage) and neuron specific enolase (a marker of neuronal damage) were detected in migraineurs (55). These degenerative processes could presumably appear in the form of white matter disintegration (reduction of FA) and cortical atrophy.

We propose that these parallel, competing mechanisms coexist, but their relative contribution is different in MWA and MWoA. However, one might see the two sub-forms of the disease a spectrum, rather than two distinct entities and hence homogenous patient groups cannot be reproducibly formed.

An alternative explanation for the structural alterations might well-be that they are not consequential but rather causal factors of the disease. Accordingly, the genetic background is different between the two subtypes. Pisanu et al. demonstrated that genetic risk factors calculated on migraine-associated single nucleotide polymorphism differ between subgroups suggesting MWA and MWoA have different genetic backgrounds that contribute to the pathogenesis (56). Even so, we cannot exclude the possibility that co-morbidities and epigenetics have an influence on migraine pathogenesis (57, 58).

A number of findings showed that the clinical expression of migraine is consistent with perivascular trigeminal activation and release of neuropeptides [calcitonin gene-related peptide (CGRP), substance P and pituitary adenylate cyclase-activating polypeptide-38 (PACAP-38)] (59–66). It was also shown that CSD is tightly connected to CGRP release (67). Several aspects of the CGRP-related trigemino-vascular functions are also abnormal in FHM1-mutant mice showing an overall hyperexcitability phenotype (68). We showed that interictal PACAP-38 concentrations were lower in migraineurs, which approached normal levels during headache (69) and this altered interictal PACAP-38 serum level correlated with the microstructural integrity of pain related brain structures (70). Whether neuropeptide concentrations are different in MWA and MWoA is still to be investigated.

Whether CSD is the initiator of all the events of migraine attack (activation of distinct brain stem nuclei, neuropeptide release at the periphery, activation, and sensitization at the level of trigeminal nociceptors) remains controversial: although that there is evidence that CSD can induce activation trigeminal nociception in animals (71), but migrainous aura can occur without headache and the pain can start during the aura onset, moreover most of the migraineurs do not experience aura phenomenon at all, which suggests that CSD alone is insufficient and non-essential for the attack. If the latter is true CSD is not the cause, but the consequence or a part of the disease (72).

Importantly, several other reasons could be pointed out behind the variable results of structural studies. For example, the headache frequency is different in MWA and MWoA (73), which means the studies should be strictly matched for clinical parameters. The time since the last and until the next headache should also be strictly monitored. Unfortunately, none of the above mentioned studies are controlled for these factors.

Importantly, we have not considered white matter hyperintensities in our review in details, but it has to be pointed out that the prevalence of these lesions are also different in the two subtypes of the diseases (74, 75). The etiology of these lesions is not entirely clear yet, but thought to be microinfarction with a numerous factors contributing to it. Neurogenic inflammation, endothelial changes, thrombocyte aggregation may play a role, and the induced oligaemia might be deepened by the CSD (76).

## Conclusion

Among primary headache disorders, migraine is a heterogeneous disease with two major subtypes. Functional imaging studies repeatedly confirmed various metrics of hyperexcitability. The results of structural imaging studies are far from being equivocal. We propose that variable contribution of parallel, competing mechanisms of maladaptive plasticity and neurodegeneration might be the reason behind the variable results. Therefore, in further research projects MWA and MWoA should be handled separately and groups should be strictly matched for clinical parameters if the two subtypes are directly compared.

## Author Contributions

ZK, DV, LV, and NS formulated the review hypothesis. NS, ZK, KK, ET, BB, BK, PF, DS, ÁP, BT, AK, JT, and DV went through on literature, collected the articles. NS, DV, and ZK wrote the manuscript.

### Conflict of Interest Statement

The authors declare that the research was conducted in the absence of any commercial or financial relationships that could be construed as a potential conflict of interest.
